# pH Dependence of Chitosan Enzymolysis

**DOI:** 10.3390/polym9050174

**Published:** 2017-05-13

**Authors:** Bi Foua Claude Alain Gohi, Hong-Yan Zeng, A Dan Pan, Jing Han, Jian Yuan

**Affiliations:** Biotechnology Institute, College of Chemical Engineering, Xiangtan University, Xiangtan 411105, Hunan, China; gohibifouaca@smail.xtu.edu.cn (B.F.C.A.G.); jessciapan017@gmail.com (A.D.P.); hanjing0528@gmail.com (J.H.); 54yuanjian@gmail.com (J.Y.)

**Keywords:** chitosan enzymolysis, parameters, kinetic, pH dependence, activation energy, inhibition

## Abstract

As a means of making chitosan more useful in biotechnological applications, it was hydrolyzed using pepsin, chitosanase and α-amylase. The enzymolysis behavior of these enzymes was further systematically studied for its effectiveness in the production of low-molecular-weight chitosans (LMWCs) and other derivatives. The study showed that these enzymes depend on ion hydronium (H_3_O^+^), thus on pH with a pH dependence fitting *R*^2^ value of 0.99. In *y* = 1.484$\left[H^{+}\right]$ + 0.114, the equation of pH dependence, when $\left[H^{+}\right]$ increases by one, *y* ($k_{0}/k_{m}$) increases by 1.484. From the temperature dependence study, the activation energy (E_a_) and pre-exponential factor (A) were almost identical for two of the enzymes, but a considerable difference was observed in comparison with the third enzyme. Chitosanase and pepsin had nearly identical E_a_, but α-amylase was significantly lower. This serves as evidence that the hydrolysis reaction of α-amylase relies on low-barrier hydrogen bonds (LBHBs), which explains its low E_a_ in actual conditions. The confirmation of this phenomenon was further derived from a similarly considerable difference in the order magnitudes of A between α-amylase and the other two enzymes, which was more than five. Variation of the rate constants of the enzymatic hydrolysis of chitosan with temperature follows the Arrhenius equation.

## 1. Introduction

Application of natural biopolymers in life sciences is useful and advantageous in several ways. The economic and environmental opportunities that come with recent developments in the concept of the “shell biorefinery” [1,2] have resulted in more attention on shellfish. For this reason, it is necessary to conduct more extensive and thorough research to identify such opportunities. There is justification for the considerable attention on the functional biopolymer, chitosan. For instance, it has wide bioavailability (from insect cell walls, fungi or marine food resources), is non-toxic, biocompatible and biodegradable [3,4]. Its utilization in medicine and the food industry is however limited due to the high molecular mass and thus high viscosity of chitosan solution. Hydrolysis of chitosan to chitosan oligomers (COS) and low-molecular-weight chitosans (LMWCs) can overcome this limitation [5]. Chitosanolysis can be done physically [6,7,8], chemically [9,10,11] or enzymatically [12,13]. In comparison with other methods, enzyme-catalyzed chitosan hydrolysis is more specific and allows for greater control of the extent of reaction. By controlling the pH, temperature and enzyme concentration, we can obtain better product size and quality [14]. This method is advantageous because it has a simple fabrication process, is environmentally friendly, commercially available and cost effective. The simple fabrication process of hydrolysis is easily controllable, simpler, more precise and does not result in the production of toxic or unusable products. The latter, coupled with the use of biodegradable waste material, contributes to it being environmentally friendly. Enzymatic hydrolysis is a lower cost alternative for the quantitative and qualitative manufacture of chemical products. The enzyme can be reused in a series of cycles of catalysis as long as the conditions remain optimum. Owing to the above, it is a more suitable technique for hydrolyzing the chitosan chain in comparison with physical and chemical catalyzation, which are expensive and sometimes inconvenient. There are no by-products, and the resulting products are biocompatible with a wide range of sizes. It is these strengths that make them usable in the fields already mentioned above. Both by-products of chitosan enzymolysis molecular weight (*M_w_*) and sequences determine the physicochemical and biological properties [15]. For example, LMWCs with average *M_w_* in the range of 9.5–8.5 kDa appear to possess stronger lyses of *Bacillus cereus*, *Escherichia coli* [16] and anti-tumor activity [17] in comparison with native chitosan. Even though chitosanase and/or chitinase are the preferred enzymes for such depolymerization processes, their usage is limited by cost, unavailability and specificity [18]. There is still a need to understand the mechanisms of the parameters at each step of enzymatic hydrolysis in order to improve on this route. This call for a cheaper alternative method of obtaining chitosan hydrolysis products has led to the testing of a number of enzymes such as pronase, pepsin, cellulases, α-amylase [16,17,18,19,20], and so on; these enzymes were found to satisfy these demands. Three of these enzymes, chitosanase, pepsin and α-amylase, were selected for further study. The reason was to compare the particularities of the hydrolysis mechanism of the specific enzyme chitosanase and commercial non-specific enzymes represented herein by pepsin and amylase.

A considerable amount of work has been published on the dependence of chitosan-hydrolyzed reactions on pH, temperature and substrate-enzyme ratio for both the specific enzyme “chitosanase” and commercial non-specific enzymes (pepsin, α-amylase, hyaluronidase, glucoamylase) [21,22,23]. The observed pH dependence and causes of variation with this parameter are however far from clear. To date, there is still not enough information about the kinetics of pH dependence on the enzymolysis process of chitosan degradation in the relevant literature. The kinetics of the substrate-enzyme ratio and temperature of the enzymolysis process of chitosan degradation have however been exhaustively described in our previous studies and by many eminent researchers [5,15,16,17,18,19,24,25]. In this context, we attempt to demonstrate the importance of pH by providing analyzed and interpreted mathematical proofs. This study does not only make observations of the reactions, but further applies the analyses and interpretations to each of the influencing factors. In addition, it confirms the primordial effect of H_3_O^+^ movement in the chitosan hydrolysis reaction and focuses on the inputs to be made at the ionic level, in order to optimize this reaction.

The aim of this work is to study the pH dependence of three different enzymes hydrolyzing chitosan. This will avail new mathematical data, which will facilitate understanding of the role and importance of pH on the overall process of chitosan enzymolysis. The investigation first focuses on optimizing the enzymolysis reaction parameters, including pH, temperature, as well as the enzyme-chitosan ratio values. Next is the evaluation of the impact of H_3_O^+^ on the process of the enzymatic hydrolysis of chitosan. This research can provide the theoretical basis and technological support for better understanding and designing the enzymatic hydrolysis of chitosan.

## 2. Materials and Methods

### 2.1. Materials

Chitosan from shrimp shells (≥91% deacetylated) and pepsin (EC 3.4.23.1, 3000–3500 units/mg protein) from porcine gastric mucosa (Amresco type A) were purchased from Sinopharm Chemical Reagent Co., Ltd. (Shanghai, China). Chitosanase from *Streptomyces griseus* (EC 3.2.1.132) and α-amylase (EC 3.2.1.1) were purchased from Sigma-Aldrich (Shanghai, China) Trading Co, Ltd. All other reagents were of analytical grade and were used without further purification. All solutions were made with redistilled and ion-free water.

### 2.2. Hydrolysis Experiments

Hydrolysis equilibrium studies were done by treating chitosan with a ≥91% degree of deacetylation, which was used as the substrate in the crude enzyme. Chitosan was dissolved in acetic acid (HAc) solution (1%, *v/v*) to make a solution of 1% (*w/w*) concentration. It was mixed with different concentrations of various enzyme solutions (50–150 mg/L of pepsin; 1–10 U of chitosanase and 40–120 U/g of α-amylase) in a stoppered bottle. It was then placed in a temperature-controlled water bath shaker (Labline, Gujarat, India). One unit (U) of crude enzymes was defined as the amount of enzyme that could liberate l μmol of reducing sugar as GlcN per min. The pH of the solution was calibrated using NaOH and HCl with a μ-362 pH meter (systronics). After completion of the reaction period (3 h), the flasks were taken out, and the reduced sugar (SRSs) concentrations were determined.

Hydrolysis studies were also performed to determine the competition between the various enzymes (chitosanase, pepsin and α-amylase). Batch experiments were performed using 1% (*w/v*) chitosan solution treated with different commercial enzymes. The contents were shaken at the optimum temperature and pH of each enzyme. The equilibrium samples were then withdrawn and centrifuged at 800 rpm for 5 min to remove the enzyme. The supernatant was stored to determine reducing sugars (SRSs) with a total organic carbon analyzer (TOC-5000A, Shimadzu, Kyoto, Japan). The tests were done in triplicate and the results recorded as an average. The SRSs’ yield was calculated as follows:

SRSs’ yield (%) = (carbon mass of SRSs)/(carbon mass of chitosan) × 100%

### 2.3. Experimental Design

In order to improve the reaction process, it is necessary to study the effect of pH, temperature and the ratio enzyme-substrate concentration on the chitosan hydrolysis reaction kinetics. To do this, determining the optimal value of each factor involved is essential. 

The pH and temperature were selected as independent variables within the recommended ranges. Unlike in our previous studies [24,25], the ‘one factor at a time’ method instead of RSM (response surface methodology) was used for optimization, because it will facilitate the kinetic study, which is our principal aim.

The experiment was initially set up to study the effect of pH and temperature (T) on chitosan enzymolysis within 6 combinations of experimental conditions; the pH ranges were 2, 3, 4, 5, 6 and 6.5. Temperature was set at 30, 40, 50, 60 and 70 °C. The pepsin-chitosan (enzyme/ substrate E/S) ratio was fixed at 1/100 (*w/w*), as reported by Roncal et al., 2007, for pepsin [26], with slight modifications. The degrees of hydrolysis under different enzyme concentrations (50, 80, 100, 120 and 150 mg/L of pepsin; 1, 3, 5, 7 and 10 U of chitosanase; 40, 60, 80, 100 and 120 U/g of α-amylase) were also investigated. These experiments were performed in a random order to avoid undesirable effects on the results.

### 2.4. Determination of pKa Values

The enzymatic hydrolysis kinetics of chitosan were determined by means of a Radiometer TTT-80 (Radiometer, Copenhagen, Denmark), pH stat at$\;\left[S_{0}]\gg [E_{0}\right]$, based on an old method with slight modifications. A Radiometer TTT lc connected to a Radiometer Titrigraph Type SBR2c (Radiometer, Copenhagen, Denmark) was used to measure the pKa values of different enzymes. The substrate was dissolved in dilute HCl (3 mL) and titrated against 0.4 M of NaOH. A second titration was carried out under identical conditions omitting the substrate. Subtraction of the first plot from the second gives a curve with a well-defined point of inflexion at the pKa value. Determination of pKa is important because it will be affected by the change in the dielectric constant of the local environment. pKa will then evolve as a function of the proton transfer mechanism of the hydrolysis medium.

### 2.5. Characterizations of Chitosan before and after Hydrolysis

Weight-average molecular weight (*M*_w_) was measured by GPC. The GPC equipment (Crown, Heppenheim, Germany) was comprised of connected columns (TSK G5000-PW and TSK G 3000-PW), a TSP P100 pump and an RI 150 refractive index indicator detector. The eluent was 0.2 M CH_3_COOH/0.1 M CH_3_COONa. The eluent and chitosan sample solutions were filtered through 0.45-um Millipore filters, maintaining the flow at 0.1 mL/min. The sample concentration was 0.4 mg/mL. Pullulan (Tosoh, Tokyo, Japan) standards were used to calibrate the column. All data provided by the GPC system were collected and analyzed using the Jiangshen workstation software package. In three hours (3 h), there was a decrease in the viscosity of the enzyme-catalyzed reaction. The reaction was continuously measured in a Cannon-Fensk (Schott Geraete, model GMBH-D65719, Mainz, Germany) capillary viscosimeter. The solutions were filtered at the lowest shear velocities permitted within the experimental error and the Newtonian plateau before measuring viscosity. The linear potentiometric method was used in the calculation of the depolymerization degree (DD) of the chitosan samples. This analysis was produced by dissolving 0.25 g of chitosan in 20 mL of HCl solution, 0.1 N. It was then filled with distilled water up to 100 mL and titrated until the chitosan solution reached approximately 6.5 pH (range of chitosan non-protonation). For the polydispersity index study, sodium alginate aqueous solution (0.1% *w/v*) was sprayed into the chitosan solution containing Pluronic F-68 (0.5% *w/v*). It was obtained at 1-, 2- and 3-h intervals of hydrolysis (0.1% *w/v*) under continuous magnetic stirring at 1000 rpm for 30 min. Interaction between the negative groups of sodium alginate and the positively-charged amino groups of chitosan (ionic gelation) resulted in the formation of nanoparticles. They were collected by centrifugation (REMI high speed, cooling centrifuge, REMI Corp., Mumbai, India) at 4 °C, at 18,000 rpm for 30 min. The sample volume used for analysis was kept constant at 5 mL to nullify the effect of stray radiations from sample to sample. The products were separated using size-exclusion chromatography (SEC) of the reaction products. This method described by Einbu et al., 2007 [27], and based on the Sørbotten et al., 2005 [28], mathematical calculations, is defined as follows. The oligomers from the neutralized reaction mixtures were separated on three Superdex^TM^30 (Amersham Pharmacia Biotech Co., Ltd. Beijing, China) columns connected in series. The columns were eluted with 0.15 M ammonium acetate at pH 4.5 and a flow rate of 0.8 mL/min. The effluent was monitored with an online refractive index (RI) detector (Shimadzu RID 20A, SHIMADZU Co., Ltd. Shanghai, China), coupled to a data logger. The relationship between detector response and mass of injected oligomer in combination with the integrals of the chromatograms from the size-exclusion chromatography below was then used to determine the molar fractions of the different oligomers.

Equation (1) is the decay in the molar concentration of tetramer [$A_{4}$], [$A_{4}$] as a function of [$A_{4}$] and the rate constants $k_{1}$ and $k_{2}$:

(1) $$\frac{d}{dt}[A_{4}]=-[A_{4}]\cdot \left(k_{1}+2k_{2}\right)$$

The change in the molar amount of trimer [$A_{3}$] in the reaction mixture is expressed in Equation (3) as the exponential function of [$A_{4}$] (Equation (1)), [$A_{3}$], and the rate constants $k_{1}$ and $k_{2}$. The first term of Equation (2) represents the formation of the trimer from the tetramer, and the second term represents the degradation of the trimer.

(2) $$\frac{d}{dt}[A_{3}]=[A_{4}]\cdot \left(k_{1}+k_{2}\right)-[A_{3}]\cdot \left(k_{1}+k_{2}\right)$$

Equation (3) expresses the change in the molar amount of dimer [$A_{2}$] with time. The first term represents the formation of the dimer from the tetramer, the second term the formation of the dimer from the trimer and the last term the degradation of the dimer.

(3) $$\frac{d}{dt}[A_{2}]=[A_{4}]\cdot 2k_{2}+[A_{3}]\cdot \left(k_{1}+k_{2}\right)-\left[A_{2}\right]\cdot k_{1}$$

The increase in the molar amount of monomer [$A_{1}$] with time is similarly given in Equation (4).

(4) $$\frac{d}{dt}[A_{1}]=[A_{4}]\cdot \left(k_{1}+k_{2}\right)+[A_{3}]\cdot \left(k_{1}+k_{2}\right)-\left[A_{2}\right]\cdot 2k_{1}$$

Equations (1)–(4) are used to determine [$A_{4}$], [$A_{3}$], [$A_{2}$] and [$A_{1}$], respectively, as a function of time with different values of $k_{2}$ and $k_{1}$.

Studies were carried out in triplicate (*n* = 3), and the standard deviation (S.D.) was recorded. 

## 3. Results and Discussion

### 3.1. Influence of the Main Factors on the Hydrolysis Process

#### 3.1.1. Effect of pH on the Chitosan Hydrolysis Process

**Figure 1:**
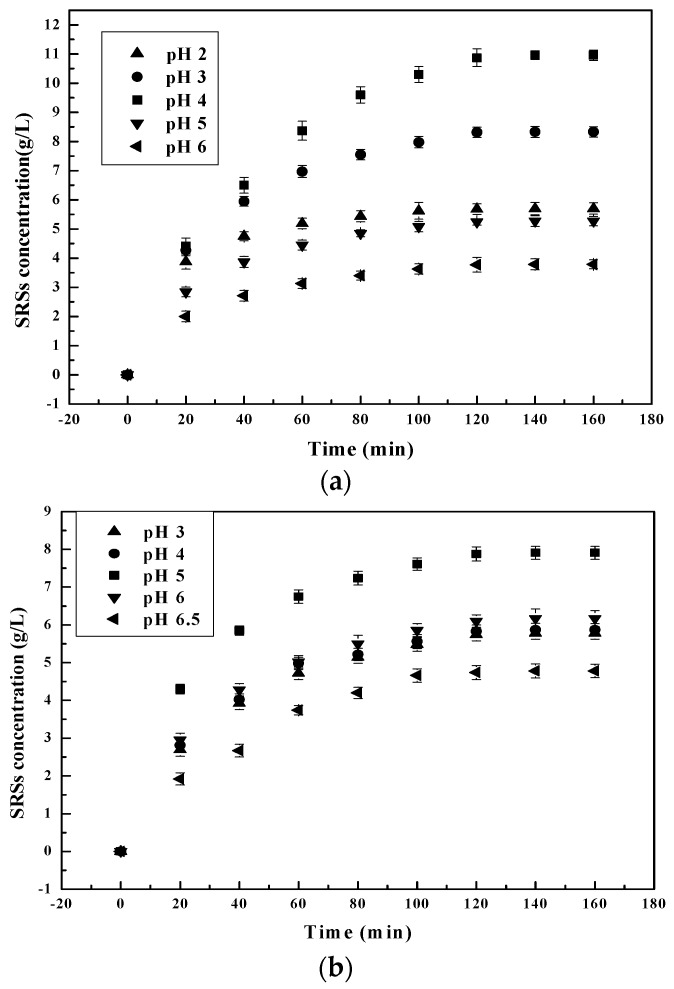

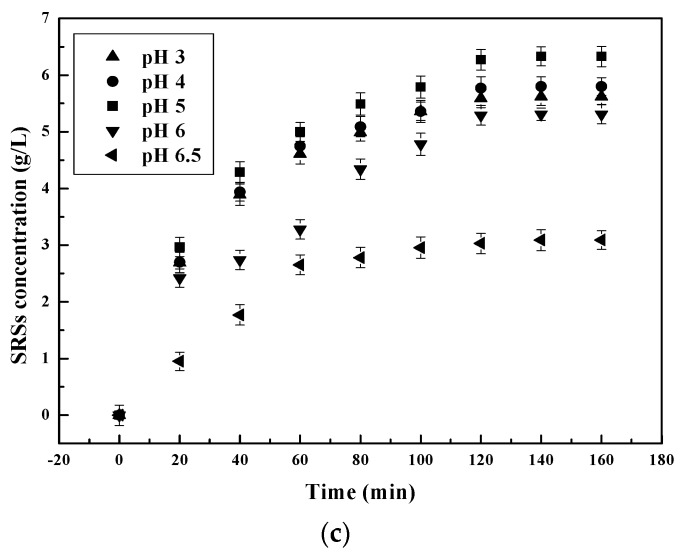
Effect of initial pH on reducing sugars’ (SRSs) production during chitosan hydrolysis at different times: (**a**) pepsin; (**b**) chitosanase; (**c**) α-amylase.

pH is one of the monitoring factors during the hydrolysis process. pH affects the activity of the functional groups of catalysts and/or substrates [29]. Experiments using solutions of chitosanase, pepsin and α-amylase with an initial enzyme/substrate ratio of 1/100 (*w/w*) were carried out to facilitate the study of the effect of pH on hydrolysis. pH solution was varied from 2–6.5 in 100-mL stoppered bottles for this batch study. The results are presented in Figure 1. 

The results reflect that an increase in pH resulted in increased SRSs’ production. After observing chitosan and its microenvironment, it was concluded that the ionic microenvironment changes the profile and pH stability of the selected enzymes. Furthermore, since chitosan is a natural basic polyelectrolyte [30], while the number of negatively-charged sites increases, the positive charge on the chitosan surface decreases faster with every increase in the pH of the solution (pH below 6.5) [31]. As the pH further increases [26], the catalytic sites of selected enzymes progressively get in contact with Glu-Glu [32], precisely on the -GlcN-GlcNAc- and -GlcNAc-GlcNAc- linkage cleavage sites of chitosan. This is due to the reduction in viscosity of the chitosan solution. This reduction in viscosity enabled the mobility of catalytic sites of enzymes that directly attacked the chitosan cleavage sites, resulting in the increase of SRSs’ production. At pH 4 for pepsin and 5 for chitosanase and α-amylase, maximum SRSs’ production was achieved as in [25,33,34] (Figure 1). Beyond the optimum pH of each selected enzyme, SSRs’ production begins to slow down as pH tends towards a neutral value of 7. SSRs’ production decreases beyond optimal pH for pepsin because it is a strong acidic protein with high enzymatic activity at lower pH values [35]. When pH goes over 4.5, the protonation of pepsin catalytic sites therefore reduces, leading to the reduction of SSRs’ production. There is easier pepsin mobility when pH is over 4.5 because of the low viscosity of the solution. This however hinders the dissolution of chitosan. In turn, it prevents contact between the active sites of the pepsin, which should have ingested carbon bonds by preferentially cleaving after the N-terminus of the chitosan oligomers. Because chitosan precipitates at pH 6, the protease activity of pepsin becomes negligible [36,37] and chitosan insoluble. The same phenomenon also occurs during the hydrolysis of chitosan by chitosanase and amylase when the pH approaches 5.5 and tends towards 6. At about pH 5.5, chitosan becomes less soluble, preventing the active sites of chitosanase and α-amylase from attacking the glycosylic [38] and glycosidic [20] bonds of the chitosan, respectively, in order to hydrolyze it.

#### 3.1.2. Effect of Temperature on the Chitosan Hydrolysis Process

**Figure 2:**
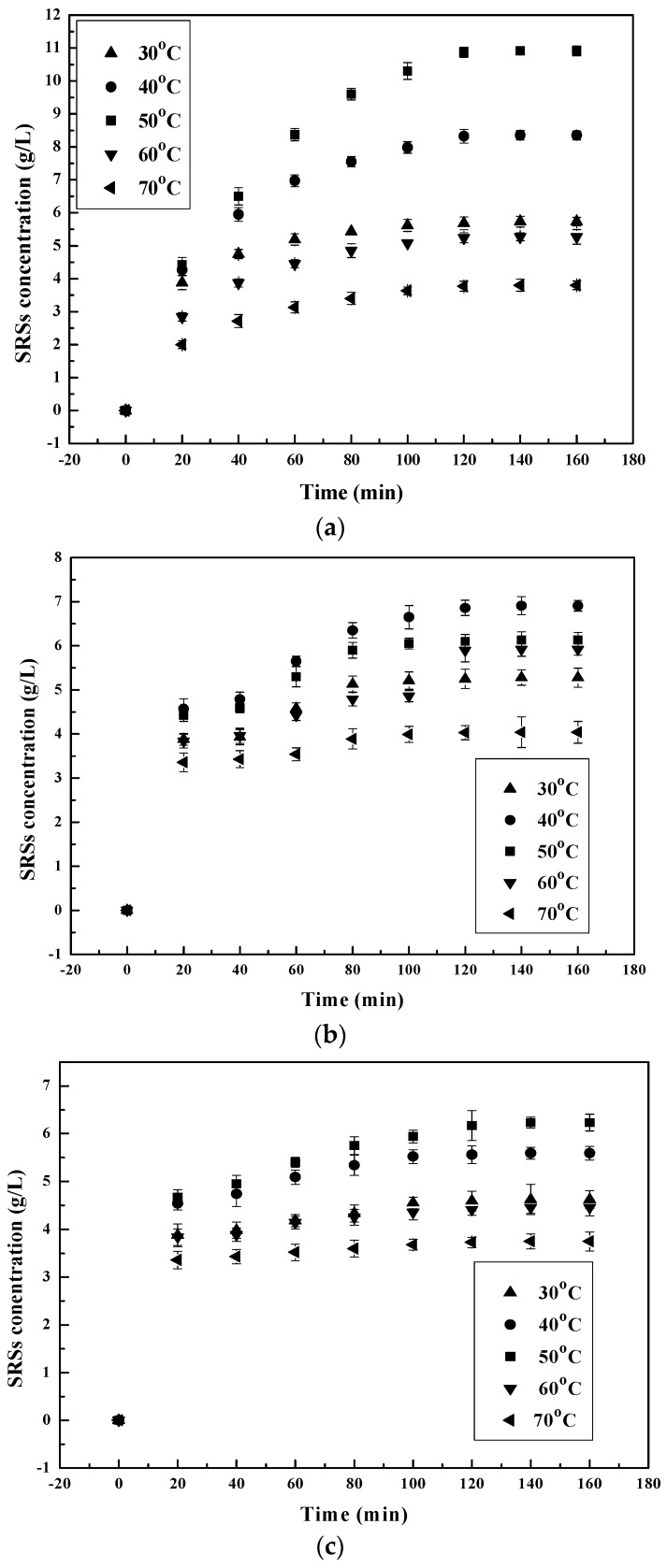
Effect of initial temperature on reducing sugars’ (SRSs) production during chitosan hydrolysis at different times: (**a**) pepsin; (**b**) chitosanase; (**c**) α-amylase.

The SSRs yield was studied at 30–70 °C to evaluate the effect of temperature on the efficiency of the chitosan hydrolysis process by the different enzymes. From the results shown in Figure 2, the increase in temperature has a significant effect on the hydrolysis of chitosan by all of the chosen enzymes. A linear increase in hydrolysis and SSRs’ production occurred at a temperature increase from 30–50 °C (Figure not shown) for pepsin and α-amylase [25,39]. When the temperature was further increased from 50–70 °C, they decreased instead. At 30, 50 and 70 degrees Celsius, the SSRs’ production of pepsin and α-amylase increased and then decreased to 5.73, 10.91 and 3.8 g/L for pepsin and 4.62, 6.23 and 3.75 g/L for α-amylase, respectively. The same phenomenon happened to chitosanase before and after 40 °C [40] with the SSRs’ production increasing and then decreasing to 5.28, 6.91 and 4.04 g/L. Thermal degradation on either or both chitosan [37] and enzyme [32] could explain the decrease in SSRs’ production yield at a temperature higher than 50 °C.

#### 3.1.3. Effect of Pepsin Concentration on the Chitosan Hydrolysis Process

**Figure 3:**
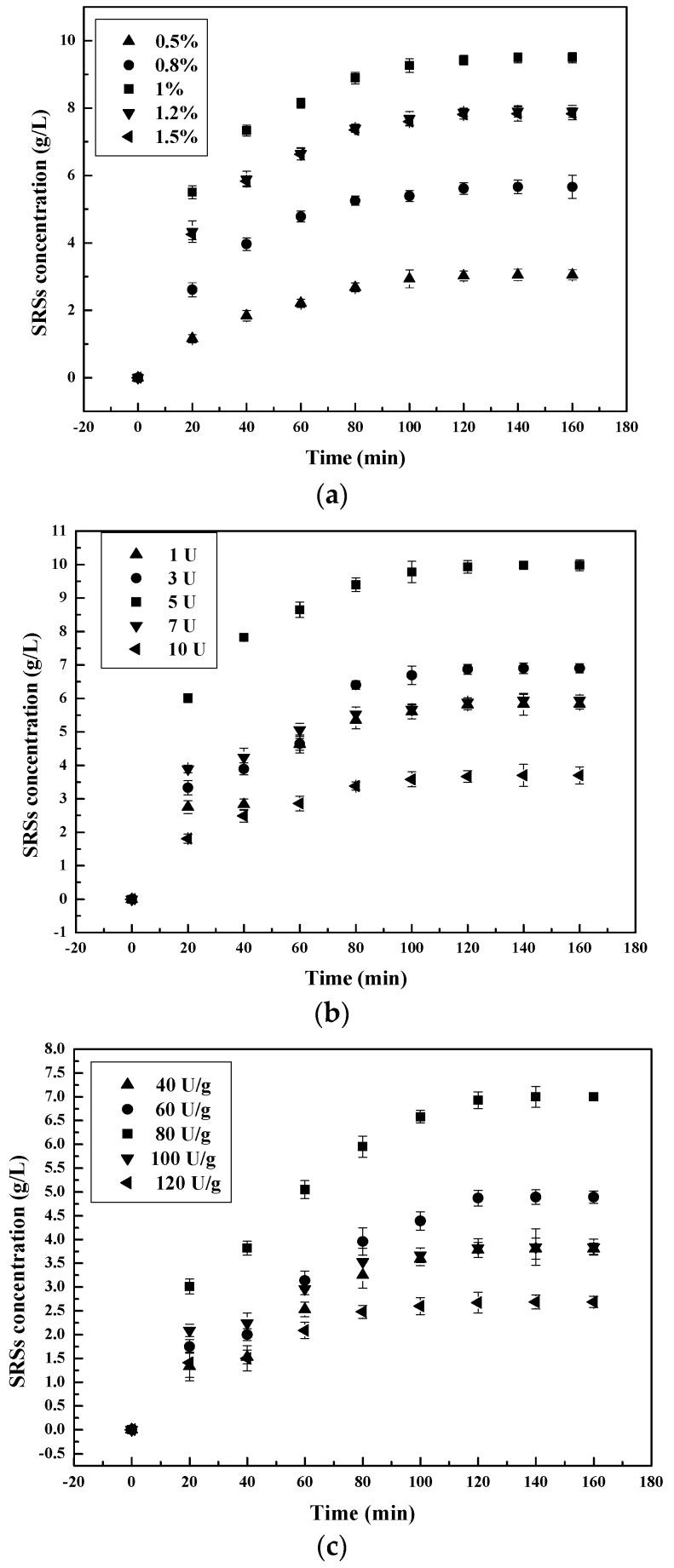
Effect of initial enzyme-substrate ratio on SRSs’ production during chitosan hydrolysis at different times: (**a**) pepsin; (**b**) chitosanase; (**c**) α-amylase.

The effect of different doses of enzymes on the hydrolysis process was studied. The results are illustrated in Figure 3.

According to this figure, after an interval of 3 h, chitosanase has the highest capacity of chitosan hydrolysis, followed by pepsin, then α-amylase. The optimum enzyme concentration for pepsin is 110 mg/L, which equals a 1.1% (*w/w*) enzyme-substrate ratio, as reported by T. Roncal et al. (2007) [25,26]. According our study, the optimum enzyme concentration was 5 U for chitosanase and 80 U/g for α-amylase, similar to previous reports. The maximum SSRs’ yield obtained was 9.98 g/L for chitosanase, 9.49 g/L for pepsin and 7.00 g/L for α-amylase.

SSRs’ production increased with the increase in enzyme concentration to the optimum concentration. Augmentation of enzyme concentration in a range above this optimum value often causes a drastic decrease of SSRs’ yield. This increase in SSRs with the increase in the dose of enzyme concentration to the optimum is probably associated with the increase in enzyme active sites. Contact and access to chitosan cleavages sites is successively increased. With the increase of all three enzyme concentrations above their optimum, the rate of hydrolysis per mass unit of enzymes decreased by ±0.53-fold. This decrease in the unit of hydrolysis with an increase in enzyme concentration is associated with the remaining unsaturated enzymes sites during the hydrolysis process.

### 3.2. Enzymolysis Kinetics

Reaction order, rate constant and activation energy are the controlling mechanisms of the process of hydrolysis used to determine kinetic models. While choosing the most suitable operating conditions for the full-scale batch process, the kinetics of chitosan enzymolysis by commercial enzyme materials is a prerequisite. The study of the kinetics of hydrolysis illustrates the rate of enzyme cleave bonds, which controls the operating time of hydrolysate formation. This rate is most important when designing the enzymolysis system and can be calculated from kinetic study. Therefore, the kinetics of chitosanase, pepsin and α-amylase onto chitosan were analyzed by using different kinetic models as presented below. This kinetic study is primarily based on pseudo second-order kinetics, because the reaction depends on the concentration of both reactants (enzymes and chitosan).

#### 3.2.1. pH Dependent Kinetic Parameters

The pH dependence was analyzed quantitatively by making a so-called Seaman’s modified equation as expressed below:

(5) $$k=k^{0}\cdot \left[H^{+}\right]\cdot e^{-E_{a}/R_{T}}$$

where k = kinetic constant (s^−1^), $k^{0}$= pre-exponential constant (s^−1^), [*H*^+^] = molar hydrogen concentration, *E_a_* = activation energy (kJ/gmol), *R* = universal gas constant (kJ/gmol·*K*), *T* = temperature (*K*). This equation assumes that H_3_O^+^ formed by the donated protons from the acid is part of the mechanism for both chitosan hydrolysis and degradation of different types of glucosamine. However, in this study, $k_{0}/k_{m}$ is used instead of k. This is owed to the very limited, almost impossible water-solubility of chitosan. It restricts the probability of getting initial rate data at substrate concentrations much greater than $k_{i}$. This led to the use of $k_{0}/k_{m}$, which is more suitable for the analysis of pH dependence.

$k_{0}/k_{m}$ is a pseudo-second-order rate constant most precisely defined from the linear transformations of the Michaelis–Menten equation. The pH dependence of this constant ($k_{0}/k_{m}$) simply reflects ionization in the pH free enzyme (and of the free substrate, if any) that affects the catalytic activity, as shown by Peller and Alberty (1959) [41].

**Figure 4:**
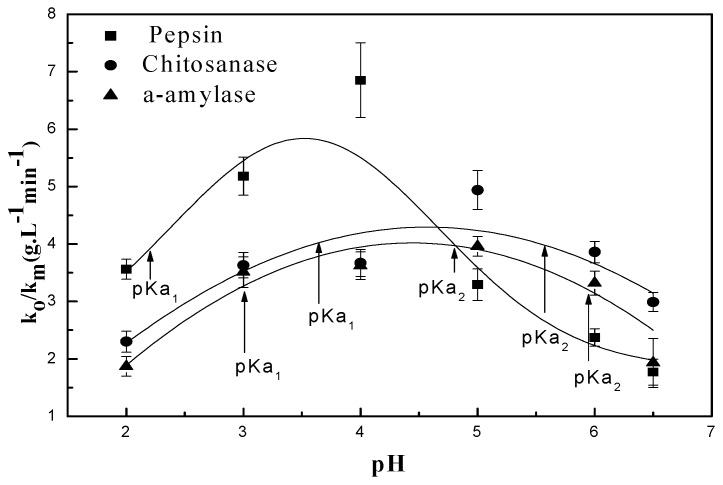
pH dependence of $k_{0}/k_{m}$ for the enzyme-catalyzed hydrolysis of chitosan: (■) pepsin; (●) chitosanase; (▲) α-amylase.

In order to determine the $k_{0}/k_{m}$ parameter, we have undertaken the experiment represented in Figure 4, the results of which are summarized in Table 1.

**Figure 5:**
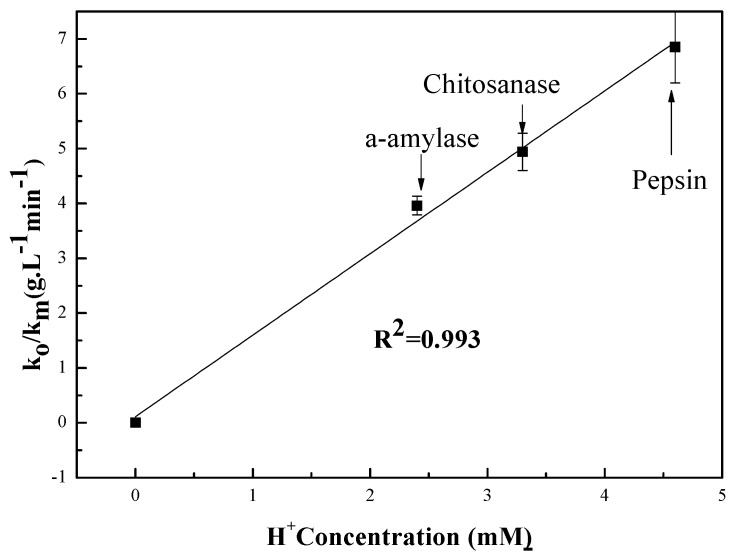
Enzymolysis of chitosan rate as a function of H^+^ concentration.

The pH-rate profile is shown in Figure 5. This figure is a plot of the hydrolysis kinetic data versus the hydrogen concentration as determined by pH measurement at room temperature. The linear fit to data results in an *R*^2^ value of 0.99 for the three tested enzymes [42]. The model for the data is *y* = 1.484[$H^{+}$] + 0.114. This indicates that for every increase of one in [$H^{+}]$, *y (*$k_{0}/k_{m}$*)* will increase by 1.484, implying a significant increase in the rate of the hydrolysis reaction when the [*H*^+^] concentration is increased. This demonstrates the overwhelming value of the contribution of H^+^ during the chitosan hydrolysis reaction. This further confirms that chitosan hydrolysis depends on hydrogen ion concentration. The data also suggest that hydrogen ions are equally effective regardless of the enzyme source [42]. Seaman’s modified equation assumes that H_3_O^+^ formed by the donated protons from the reaction environment is part of mechanism for both chitosan and enzyme types [43]. It is also known that proton transfer mechanisms play an important role in enzymatic reactions [44]. This involves using the pH of the different solutions to determine the rates of both hydrolysis and polysaccharide “chitosan” degradation.

#### 3.2.2. Temperature Dependence of Kinetic Parameters

**Figure 6:**
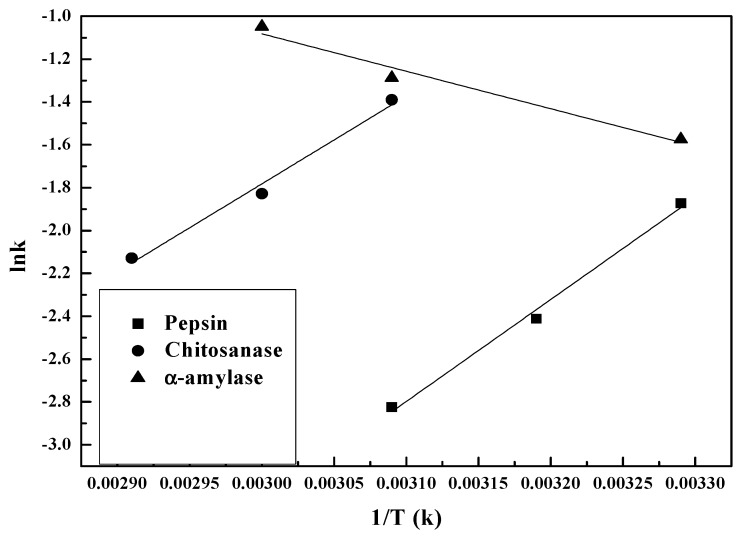
Arrhenius diagram showing the temperature dependence of chitosan hydrolysis rate constants: ln k vs. 1/T (K): (■) pepsin; (●) chitosanase; (▲) α-amylase.

Figure 6 shows plots of log enzymolysis rate vs. 1/T over temperatures ranging from 30–70 °C. The Arrhenius relationship can be written as:

(6) $$\ln\left(k\right)=\mathrm{lnA}-\left(\frac{\mathrm{Exp}}{R}\right)\left(\frac{1}{T}\right)$$

where A is a pre-exponential term, Exp denotes the experimental activation energy (kJ mol^−1^), R is 8.3144 J mol^−1^ K^−1^ and T is the temperature (K). Equation (6) argues that for reactants to transform into products, they must first acquire a minimum amount of energy at an absolute temperature T. This energy is called the activation energy and is the formula for the temperature dependence of the reaction rate constant.

The subset of experiments on various enzymes conducted between 30 and 70 °C and at different optimum pH and enzyme-substrate ratios displayed a linear relationship between log rate and inverse temperature (K^−1^). 

The increase in temperature from 30–50 °C led to an increase in log k for chitosanase and pepsin, but a decrease for α-amylase. Towards 50 °C, the hydrolysis rates reached the maximum for pepsin and α-amylase, then decreased with 1/T (K). Note that at the same temperature, no previous hydrolysis rate data were obtained to compare. 

Table 2 indicates that for both pepsin and chitosanase, the activation energies are identical to the margin of error [45]. It also shows that temperature has a greater impact on the kinetic rate of chitosan depolymerization in the presence of chitosanase and pepsin. The activation energy of hydrolysis is 15.03 kJ/mol for pepsin and 12.82 kJ/mol for chitosanase. α-amylase clearly has the lowest experimental activation energy of 5.43 kJ/mol for hydrolysis between 50 and 70 °C at pH 5. The considerable difference implies the existence of an alternative mechanism, low-barrier hydrogen bonds LBHBs [46]. This could be the stronger mechanism, thus reducing the activation energy required during chitosan hydrolysis by α-amylase. Moreover, the considerable difference (>5) is also reflected in the order magnitudes of A between α-amylase and the other two enzymes, further confirming the contribution of LBHBs. The variation of the rate constants of the enzymatic hydrolysis of chitosan with temperature as shown in Figure 6 follow the Arrhenius equation since all of the fitting *R*^2^ are above 0.95. This Arrhenius plot of chitosan hydrolysis kinetics confirms that hydrogen ions in the aqueous form H_3_O^+^ are responsible for chitosan hydrolysis independent of the enzyme source [42]. All of the above demonstrates the omnipresence of H_3_O^+,^ and therefore the key role of pH in the enzymolysis process of the degradation of chitosan.

#### 3.2.3. Hydrolysis Process Dependence on Enzyme Concentration

Application of the models of heterogeneous catalytic reactions for the description of the kinetics of enzymatic hydrolysis was enabled by the heterogeneous nature of the system chitosan-enzyme (pepsin, chitosanase and α-amylase). The effect of the enzyme complex concentration on the hydrolysis process of the kinetics followed. We used 50, 80, 100, 120 and 150 mg/L of pepsin, 1, 3, 5, 7 and 10 U of chitosanase and 40, 60, 80, 100 and 120 U/g of α-amylase solutions of enzyme concentration, referred to as the mass. The experiments were carried out at different predetermined optimum values of the various parameters (pH, T) of the enzymes. The reducing sugar amount, SRSs, was obtained during the course of enzymatic hydrolysis, which varied with time. Figure 3 and the Supplementary Materials illustrate the corresponding curves representing different concentrations for each of the enzymes. 

The increase of enzyme concentration led to increased amounts of SRSs. That effect is better outlined at 110 mg/L, 5 U and 82 U/g respectively for pepsin, chitosanase and α-amylase. The kinetic parameters for the data from the present study were calculated on the basis of first- and second-order models (Figure 3 and Supplementary Materials). Results are in Table 3. 

From comparison of the two models, we observed that the second-order model (*R*^2^ 0.997~0.999) was more suitable for describing the enzymolysis process of chitosan based on a higher *R*^2^. The results show that all of the correlation coefficients *R*^2^ in the two models are above 0.950. However, only the second-order model is applicable, considering the type of the hydrolysis reaction of chitosan. In the second-order model, the theoretical values (*Q*_e_) were in good agreement with the corresponding experiment values (*Q*_e,exp_). Hydrolysate SRSs’ concentration increased with enzyme concentration up to the maximum *Q*_e_ value of 110 mg/L, 5 U and 82 U/g for pepsin, chitosanase and α-amylase, respectively. It then steadied with the further increase in enzyme concentration. The hydrolysis rate constant *k* exhibited a similar trend. The implication from this is that chitosan has an inhibitory effect on enzyme hydrolysis activity at high concentrations (> about the optimum value). It stayed unchanged in the case of enzyme concentrations above the optimum concentration. This indicates some hindrances during the course of the process, most probably determined by enzyme saturation of the substrate [47]. The maximum value of the hydrolysis rate for each enzyme concentration is achieved at the equilibrium stage after 120 min and gradually lowers with the increase in time. Over 75% SRSs are produced within the first 2 h for each initial enzyme concentration. The slowdown of the hydrolytic rate can be strongly attributed to enzyme deactivation, chitosan/polymers/ash against enzyme adsorption and substrate recalcitrance [48]. 

In the second-order model, the constant *k* had a specific hydrolysis rate (*v*) and was used to calculate the initial hydrolysis rate *h*, at *t*→0, as follows in Equation (7).

*h* = *kQ*_e_^2^ (7)

where *Q*_e_ was the SRSs’ concentration in the reaction solution at equilibrium. Initial hydrolysis rate *h* increased with enzyme concentration to the optimum. It then decreased due to the inhibitory effect of the ratio as it was further increased. The results from this experiment argue that the process of enzymolysis should be operated at an enzyme-chitosan ratio below 110 mg/L, 5 U and 82 U/g for pepsin, chitosanase and α-amylase, respectively, to avoid enzyme inhibition

### 3.3. Relationship Between pH and Degradation Mechanisms of Each Enzyme

**Figure 7:**
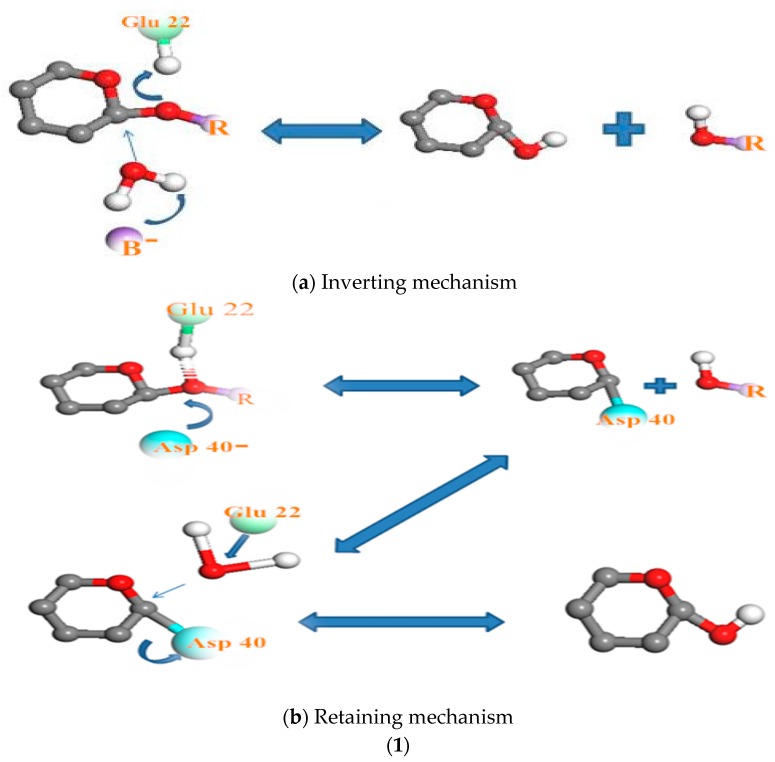

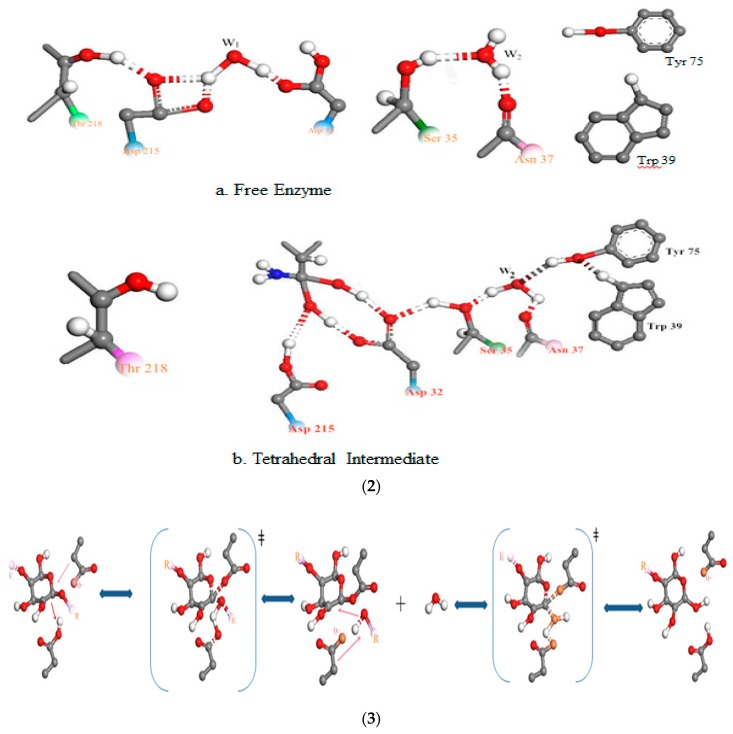
The relationship between pH and degradation mechanisms at the active site of each enzyme; (**1**) chitosanase mechanisms: (**a**) inverting mechanism and (**b**) retaining mechanism of cleaving glycosylic bonds proposed by Thadathil, N. and Velappan, S.P (2014) [38]; (**2**) mechanism of the pepsin-like family of aspartic peptidases proposed by Andreeva N.S., Rumsh L.D. (2001) [62]. (**3**) Mechanism of α-amylase cleaving glycosidic bonds proposed by MacGregor, E.A., et al. (2001) [54]. See the Supplementary Materials (relationship between pH and degradation mechanisms at the active site of each enzyme) for more detailed explanations.

The activity and stability of the enzyme are closely related to the prevailing pH of its environment [49]. The mechanism of degradation of the enzymes studied, regardless of specific or commercial non-specifics enzymes, depends on pH, Figure 7. In other words, it depends on the displacement of ions in the aqueous solution, as we have already illustrated above.

The relationship between pH and the mechanism of chitosanase degradation is better highlighted by Thadathil and Velappan (2014) [38]: the “retaining mechanism” and the “inverting mechanism”. With the retaining mechanism, the glycosidic oxygen is protonated by Glu22-H, and Asp40 provides nucleophilic assistance to aglycon departure (or vice versa). More explicitly, the retaining glycosidases catalyze the hydrolysis via a two-step double-displacement mechanism, with one of the two essential amino acid residues functioning as a nucleophile, and the other as a general acid/base. However, in the second mechanism, the inverting glycosidases follow a one-step single displacement mechanism, with the assistance of a general acid and a general base. The general base polarizes a water molecule to develop a stronger nucleophile for attacking the anomeric carbon, whereas the general acid protonates the glycosidic oxygen to accelerate the reaction. In this particular enzyme, Glu22 was found to act as a proton donor, in cooperation with Asp40. In so doing, the water molecule to attack the anomeric carbon of the glucosamine residue in the substrate was activated [50]. For pepsin, the stability of the enzyme strongly depends on the acidic pH of the medium. Acid medium (pH <6, medium capable of donating a hydron (proton or hydrogen ion H^+^)) is required to convert inert pepsinogen (the precursor of the active pepsin enzyme) into active pepsin. The released pepsin initiates digestion through proteolysis [51]. Once converted, the activated pepsin continues the autocatalytic process sustaining the cascade in the absence of acid [52]. Pepsin is a large bilobed concave molecule, the concavity being occupied by the detachable pro-part. When in contact with medium capable of donating a proton or hydrogen ion H^+^, the pro-part detaches, exposing the concavity. This is the active site for enzyme action. The enzyme attaches to its substrate at this point and cleaves it [49]. Hydrophobic interactions are the most important interactions in protein conformation [53]. These types of interactions maximize hydrogen bonding between molecules of water and minimize the area of contact between water and nonpolar molecules. The result is primordial intervention of pH in the activation of the enzymes’ mechanism. The catalytic mechanism of α-amylase is also carried out in two steps. The first stage involves displacement of protons as in the first two mechanisms (of pepsin and chitosanase). According to MacGregor et al. (2001) [54], this catalytic reaction is proceeded by a double displacement mechanism. An acid group on α-amylase protonates the glycosidic oxygen, and the catalytic nucleophile attacks at C1; consequently forming an oxocarbenium ion-like transition state during the first displacement [55]. The second stage proceeds via an oxocarbenium ion-like transition state, which promotes the attack of the incoming molecule ROH on the formation of the covalent intermediate. This results in a second transition state and thus hydrolysis or transglycosylation products [56,57].

### 3.4. Characterizations of the Enzymolysis Products

**Figure 8:**
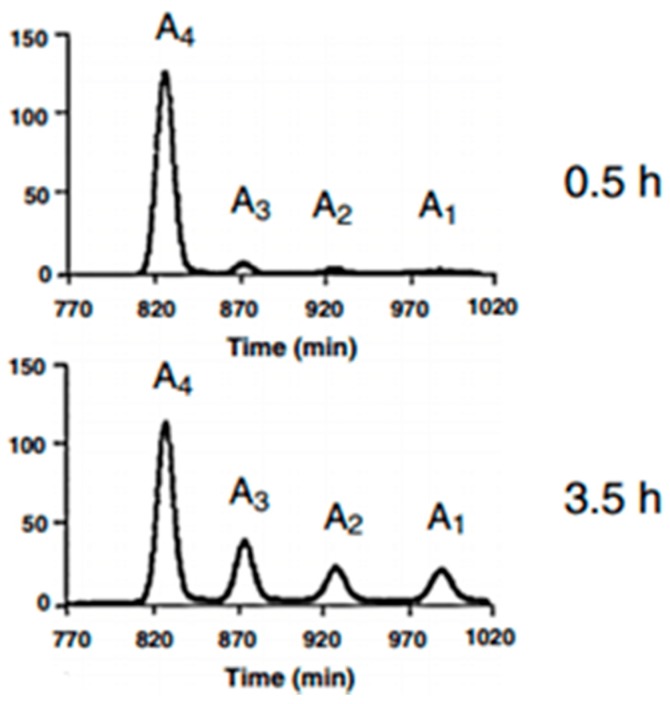
Illustration of the size-distribution of the molar fraction after hydrolysis of chitosan (diagram from Einbu et al., 2007 [27]). The gel filtration chromatograms (SuperdexTM30) display peaks of the tetramer (A4), trimer (A3), dimer (A2) and monomer (A1)).

Characterization of the products will focus on the products resulting from hydrolysis by chitosanase and α-amylase. Those from the hydrolysis of chitosan by pepsin have already been widely described in our previous study [25]. Characterization of enzymolysis products is essential in industrial applications. The depolymerization degree (*DD*), molecular weight (*M_w_*), viscosity, polydispersity index and size-distribution of the products are very important parameters in characterizing the products and in the enzymolysis process. Methods of determining these parameters are exhaustively described in Section 2.5. In conclusion, in the presence of optimal conditions for hydrolysis, the chitosan chain is submitted to degradation due to the efficiency of the different parameters and the prolonged time necessary for obtaining the advanced depolymerization. Enzymes cause fast viscosity decreases and reduce end releases. LMWC products with different *M_w_* and chains with different lengths and compositions are attributed to the differences in time. The main *DD* of chitosan’ (before hydrolysis) with a decrease in molecular weight indicates that enzymes selectively cleave preferential bonds [58]. After 2 h of enzymolysis, the average *M_w_* decreased from 300 to 126 × 10^3^ Da with enzymolysis time, whereas it decreased to 0.004 × 10^3^ Da with chitosanase and α-amylase treatments after 3 h. After 3 h of hydrolysis, the polydispersity index (PDI = *M*_w_/*M*_n_) was studied, and monomers were detected in the reducing ends after chitosanase and α-amylase treatments. Following the neutralization of the reaction mixture [59], the resulting products from the enzymolysis of chitosan by chitosanase and α-amylase had soluble and insoluble fractions. The standard samples were detected by HPLC. The results presented that the enzymolysis by chitosanase and α-amylase gradually and totally hydrolyzes chitosan, which could produce large quantities of short- and medium-chain oligosaccharides (monomer, N-acetylglucosamine, D-glucosamine, dimer, trimer and tetramer and pentamer). This indicates that the chitosanase and α-amylase are able to split not only the β-1,4-glycosidic linkages of GlcN-GlcN GlcN-GlcNAc, but also those between 2 GlcN (GlcN-GlcN) and/or GlcN-GlcNAc. There were COS, LMWCs and monomers in different proportions depending on the time of hydrolysis, thus confirming [26,39,60]. The *DD*, *M_w_*, viscosity average and polydispersity index of chitosan degraded by chitosanase and α-amylase are listed in Table 4. These findings can be interpreted as a confirmation that chitosan was degraded into smaller molecular weight units. These chromatograms from Einbu et al., 2007 [27], Figure 8, show well-resolved peaks of chitosan-hydrolyzed products, which could be accurately quantified into molar fractions. As in the diagram, the heavy chain of chitosan is disaggregated into smaller molecular fractions ranging from chitooligosaccharides to monomers via LMWC as a function of time. 

In contrast to the mechanism of hydrolysis of pepsin, which does not lead to the monomer fraction after only 3 h of incubation [25,26], chitosanase and α-amylase result in a small fraction of monomers as noted in Table 4 in the same time laps [20,34,38,61].

## 4. Conclusions

Pepsin, chitosanase and α-amylase were used to hydrolyze chitosan. Mechanical and kinetic explanations of the importance of pH showed that chitosan can be hydrolyzed at a pH very close to 7. pH dependence of the enzymes explains the hydrolysis process. However, in order to optimize the process of chitosan hydrolysis, it was important to highlight all of the influencing factors. The key role of H_3_O^+^ during this process was highlighted by the different parameters in the pH and temperature dependence equations. The temperature kinetics was fitted by the Arrhenius model. Higher temperature (>40 for chitosanase; >50 for pepsin and α-amylase) generally resulted in the decrease of SSRs. The kinetic mechanism of chitosan hydrolysis by the enzymes responds to a model of inhibition by the temperature and substrate-enzyme concentration. *h* decreased with the enzyme-substrate concentration ratio above the optimum enzyme concentration, then decreased due to the inhibitory effect of the ratio as it further increased. It was found that enzymolysis could effectively help to maximize the use of chitosan and has both economic and environmental value in the related industrial applications.

## Figures and Tables

**Table 1:** pH independence kinetic parameters $k_{0}/k_{m}$ and pKa of chitosan enzymolysis.

Enzymes	*M*_w_ (kDa)	$k_{0}/k_{m}$ (gL^−1^ min^−1^)	pKa		
			1	2	3
α-amylase	55	3.96	3	6	-
Chitosanase	47	4.94	3.59	5.54	-
Pepsin	34.64	6.85	2.22	4.81	-

**Table 2:** Arrhenius kinetic parameters A (pre-exponential factor) and E_a_ (activation energy) of chitosan enzymolysis.

Enzyme	A/min^−1^	E_a_/kJ·mol^−1^	*R*^2^
Pepsin	5.8 × 10^7^	15.028	0.986
Chitosanase	1.02 × 10^5^	12.822	0.982
α-amylase	66.282	5.439	0.956

**Table 3:** First- and second-order kinetic parameters *k*_1_ (first-order hydrolysis rate), *k*_2_ (second-order hydrolysis rate), *h* (initial hydrolysis) and *R*^2^ of chitosan enzymolysis.

**Pepsin**					
**Ratio Sub-Enzyme**	**First-Order** *Qt* = *Qe*(1 − exp( − *k*_1_t)) *		**Second-Order** t/*Qt* = 1/(*k*_2_*Qe*^2^) + t/*Qe* *		
	***k*_1_ (1/min)**	***R*^2^**	***k*_2_ (L/(g·min))**	***h* (g/(L·min))**	***R*^2^**
0.5%	0.030	0.999	0.00488	4.168	0.992
0.8%	0.039	0.995	0.00484	6.943	0.996
1%	0.036	0.995	0.00483	10.840	0.999
1.2%	0.035	0.996	0.00487	9.226	0.998
1.5%	0.021	0.996	0.00486	9.163	0.998
**Chitosanase**					
**Enzyme Concentration**	**First-Order** *Qt* = *Qe*(1 − exp( − *k*_1_t)) *		**Second-Order** t/*Qt* = 1/(*k*_2_*Qe*^2^) + t/*Qe* *		
	***k*_1_ (1/min)**	***R*^2^**	***k*_2_ (L/(g·min))**	***H* (g/(L·min))**	***R*^2^**
1 U	0.022	0.964	0.0028	7.841	0.961
3 U	0.042	0.994	0.00247	9.157	0.966
5 U	0.042	0.972	0.00508	11.282	0.999
7 U	0.028	0.992	0.00913	6.585	0.989
10 U	0.022	0.964	0.00695	4.560	0.994
**α-Amylase**					
**Ratio Sub-Enzyme**	**First-Order** *Qt* = *Qe*(1 − exp( − k_1_t)) *		**Second-Order** t/*Qt* = 1/(k_2_*Qe*^2^) + t/*Qe* *		
	***k*_1_ (1/min)**	***R*^2^**	***k_2_*(L/(g·min))**	***h* (g/(L·min))**	***R*^2^**
40 U/g	0.014	0.97851	0.00188	6.218	0.968
60 U/g	0.021	0.98822	0.00137	8.078	0.976
80 U/g	0.026	0.96801	0.00209	9.532	0.990
100 U/g	0.026	0.96857	0.00614	4.812	0.980
120 U/g	0.015	0.97291	0.00826	3.400	0.972

* *Q*_t_ and *Q*_e_ are the SRS concentration at *t* and equilibrium, respectively; *k*_1_ and *k*_2_ are the rate constants of thefirst-order and second-order models, respectively.

**Table 4:** Characteristics of chitosan before and after enzymolysis.

Source	*M*_w_ (×10^3^)	DD (%)	Viscosity Decrease (%)	Yield (%)
Native	300	-	-	-
CH_2_	186–126	65.50 _a_	55 _a_	-
		78.9 _b_	75 _b_	-
CH_3_	110–80	86.00 _a_	77 _a_	-
		90 _b_	88 _b_	-
COS _2_	90–85	-	-	86.34 _a_
				55.00 _b_
COS _3_	64–47	-	-	77.30 _a_
				42.40 _b_
LMWC _2_	20–15	-	-	13.66 _a_
				41.80 _b_
LMWC _3_	10–1.2	-	-	19.70 _a_
				53.70 _b_
Monomers _2_	0.004–0.01	-	-	1.0 _a_
				2.6 _b_
Monomers _3_	0.004–0.01		-	3.0 _a_
				4.10 _b_

2: after 2 h; 3: after 3 h; CH_2_: chitosan hydrolyzed after 2 h; CH_3_: chitosan hydrolyzed after 3 h; monomers: sum of GlcN and GlcNAc; COS: chitosan oligosaccharides; LMWC: low-molecular-weight chitosan; _a_: chitosanase values; _b_: α-amylase values.

## Supplementary Materials

The following are available online at www.mdpi.com/2073-4360/9/5/174/s1, Figure S1: Figures supporting the first and second order kinetic analysis; A. pH, B. temperature and C. enzyme with A.1, B.1 and C.1 pepsin, A.2, B.2 and C.2 chitosanase. and A.3, B.3 and C.3 α-amylase in the context of kinetic study. Table S1: First and second orders kinetic parameters *k_1_* (first order hydrolysis rate); *k_2_* (second order hydrolysis rate) *h* (initial hydrolysis) and *R^2^* of chitosan enzymolysis; A.pH, B. temperature and C. enzyme, with 1. pepsin, 2. chitosanase and 3. α-amylase in the context of kinetic study. Text S1: Relationship between pH and degradation mechanisms at the active site of each enzyme.
