# Supplementary material for: pH Dependence of Chitosan Enzymolysis
Source: Polymers (Basel). 2017 May 13;9(5):174. doi: 10.3390/polym9050174 (PMC6432485; doi:10.3390/polym9050174)
Supplement: Supplementary file 1 [file polymers-09-00174-s001.pdf]

Supplementary Materials: pH Dependence of Chitosan Enzymolysis

Table S1: First and second orders kinetic parameters k_1 (first order hydrolysis rate); k_2(second order hydrolysis rate); h (initial hydrolysis) and R^2 of chitosan enzymolysis; A.pH, B. temperature and C. enzyme, with 1. pepsin, 2. chitosanase and 3. α-amylase in the context of kinetic study.

Table A. pH kinetics.

A.1. Pepsin

pH	First-order $Qt = Qe(1–\exp(–k1t))$*		Second-order $t/Qt= 1/(k2Qe2)+t/Qe$*		
	k_1 (1/min)	R^2	k_2 (L/(g·min))	h (g/(L·min))	R^2
2.0	0.05319	0.99378	0.01344	6.21335	0.99937
3.0	0.03228	0.99649	0.00387	9.93123	0.99825
4.0	0.0224	0.99753	0.0015	14.63602	0.99474
5.0	0.0348	0.99588	0.007	6.17049	0.99855
6.	0.03288	0.99374	0.0089	4.4723	0.99847

A.2. Chitosanase

pH	First-order $Qt = Qe(1–\exp(–k1t))$*		Second-order $t/Qt= 1/(k2Qe2)+t/Qe$*		
	k_1 (1/min)	R^2	k_2 (L/(g·min))	h (g/(L·min))	R^2
3.0	0.02839	0.99803	0.00441	7.12559	0.99785
4.0	0.02991	0.99738	0.00475	7.14079	0.9967
5.0	0.03041	0.99656	0.00482	9.22024	0.99875
6.0	0.0295	0.99743	0.00445	7.50791	0.99851
6.5	0.02179	0.99252	0.00321	6.51282	0.98718

A.3. α-amylase

pH	First-order $Qt = Qe(1–\exp(–k1t))$*		Second-order $t/Qt= 1/(k2Qe2)+t/Qe$*		
	k_1 (1/min)	R^2	k_2 (L/(g·min))	h (g/(L·min))	R^2
3.0	0.02947	0.9973	0.00486	6.85356	0.99806
4.0	0.02851	0.99648	0.00445	7.10552	0.99794

5.0	0.02793	0.99391	0.004	7.73973	0.99841
6.0	0.01778	0.96332	0.00221	7.51235	0.96864
6.5	0.02171	0.98306	0.0046	4.30341	0.97029

Table B. Temperature kinetics.

B.1 Pepsin

Temperature	First-order $Qt = Qe(1–exp(–k1t))$*		Second-order $t/Qt= 1/(k2Qe2)+t/Qe$*		
	k_1 (1/min)	R^2	k_2 (L/(g·min))	h (g/(L·min))	R^2
30	0.03215	0.99640	0.01320	6.23550	0.99960
40	0.02260	0.99733	0.00384	9.95274	0.99844
50	0.03480	0.99588	0.00152	14.55741	0.99417
60	0.03269	0.99355	0.00700	6.17050	0.99855
70	0.05279	0.99329	0.00880	4.486153	0.99866

B.2. Chitosanase

Temperature	First-order $Qt = Qe(1–exp(–k1t))$*		Second-order $t/Qt= 1/(k2Qe2)+t/Qe$*		
	k_1 (1/min)	R^2	k_2 (L/(g·min))	h (g/(L·min))	R^2
30	0.05288	0.96012	0.01215	6.63893	0.98445
40	0.04044	0.95861	0.00814	6.36026	0.94490
50	0.05130	0.96466	0.04068	4.15027	0.98874
60	0.03800	0.90416	0.01486	5.69421	0.99000
70	0.08720	0.97249	0.00744	7.67671	0.98074

B.3. α-amylase

Temperature	First-order $Qt = Qe(1–exp(–k1t))$*		Second-order $t/Qt= 1/(k2Qe2)+t/Qe$*		
	k_1 (1/min)	R^2	k_2 (L/(g·min))	h (g/(L·min))	R^2
30	0.09263	0.97955	0.02667	5.78941	0.99463
40	0.08005	0.98081	0.01587	6.52240	0.99048

50	0.06276	0.96903	0.05153	4.51772	0.99526
60	0.10213	0.98537	0.08950	3.77807	0.99737
70	0.12121	0.99183	0.04028	4.71309	0.99288

Table C. Enzymes kinetics.

C.1. Pepsin

Ratio sub-enzyme	First-order $Qt = Qe(1–exp(–k1t))$*		Second-order $t/Qt= 1/(k2Qe2)+t/Qe$*		
	k_1 (1/min)	R^2	k_2 (L/(g·min))	h (g/(L·min))	R^2
0.5%	0.02992	0.99964	0.00488	4.16755	0.99207
0.8%	0.03944	0.99484	0.00484	6.94279	0.99475
1%	0.03566	0.99531	0.00483	10.83961	0.99855
1.2%	0.03551	0.99642	0.00487	9.22588	0.99757
1.5%	0.02134	0.99594	0.00486	9.16266	0.99751

C.2. Chitosanase

Enzyme concentration	First-order $Qt = Qe(1–exp(–k1t))$*		Second-order $t/Qt= 1/(k2Qe2)+t/Qe$*		
	k_1 (1/min)	R^2	k_2 (L/(g·min))	h (g/(L·min))	R^2
1 U	0.0222	0.96438	0.0028	7.84116	0.96076
3 U	0.04179	0.99389	0.00247	9.15687	0.96626
5 U	0.04229	0.97161	0.00508	11.28241	0.99859
7 U	0.02821	0.99202	0.00913	6.58533	0.98878
10 U	0.02215	0.96402	0.00695	4.56011	0.99417

C.3. α-amylase

Enzyme concentration	First-order $Qt = Qe(1–exp(–k1t))$*		Second-order $t/Qt= 1/(k2Qe2)+t/Qe$*		
	k_1 (1/min)	R^2	k_2 (L/(g·min))	h (g/(L·min))	R^2
40 U/g	0.01397	0.97851	0.00188	6.21758	0.96763
60 U/g	0.02063	0.98822	0.00137	8.0777	0.97585
80 U/g	0.02626	0.96801	0.00209	9.53159	0.98881
100 U/g	0.02557	0.96857	0.00614	4.81184	0.97649
120 U/g	0.01475	0.97291	0.00826	3.39527	0.97179

Figure S1: Figures supporting the first and second order kinetic analysis; A. pH, B. temperature and C. enzyme with A.1, B.1 and C.1 pepsin, A.2, B.2 and C.2 chitosanase. and A.3, B.3 and C.3 α-amylase in the context of kinetic study.

Figure A. pH kinetics.

A.1. Pepsin

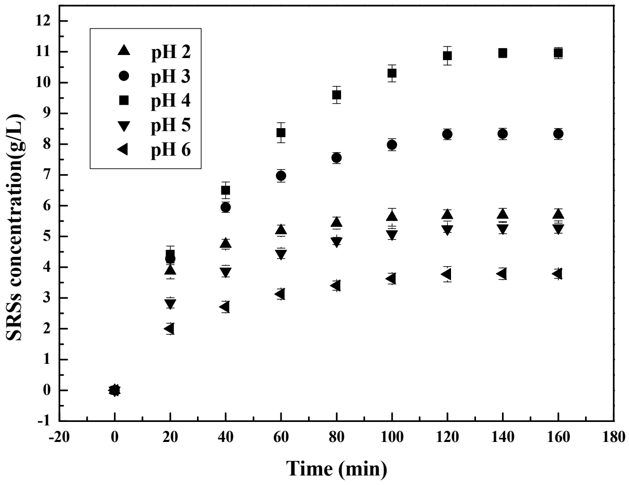

A.2. Chitosanase

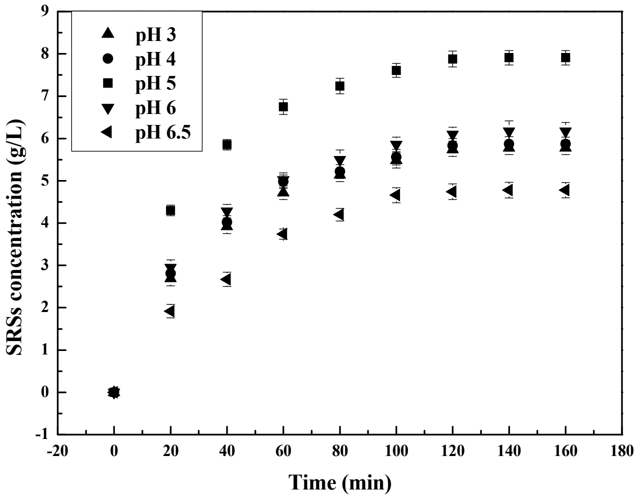

A.3. α-amylase

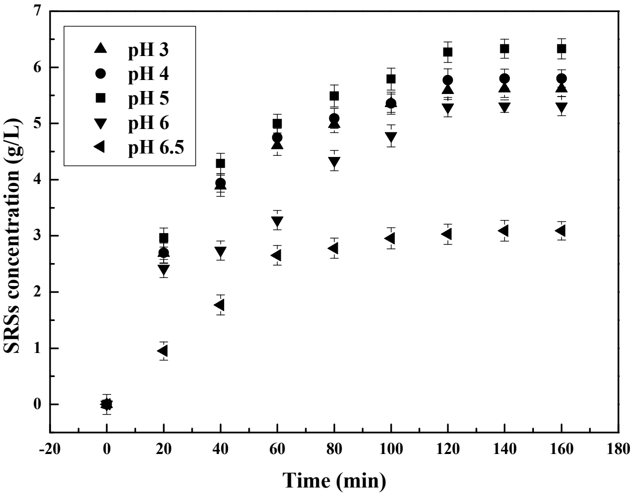

pH 3
pH 4
pH 5
pH 6
pH 6.5
SRSs concentration (g/L)
Time (min)
0
1
2
3
4
5
6
7
-20
0
20
40
60
80
100
120
140
160
180

Figure B. Temperature kinetics.

B.1. pepsin

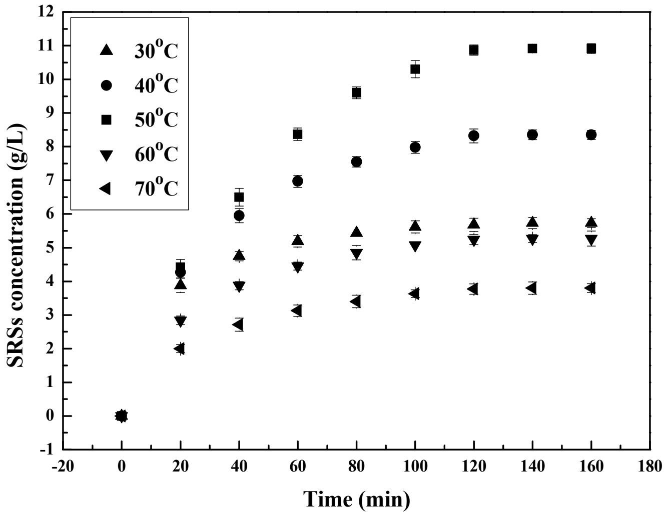

B.2. Chitosanase

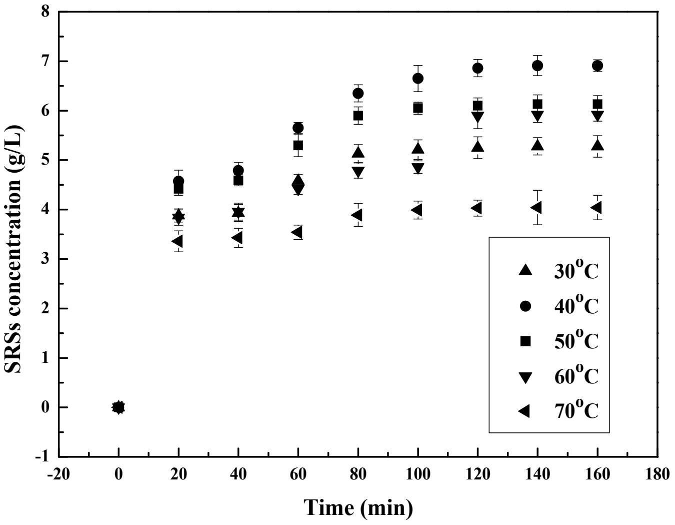

B.3. α-amylase

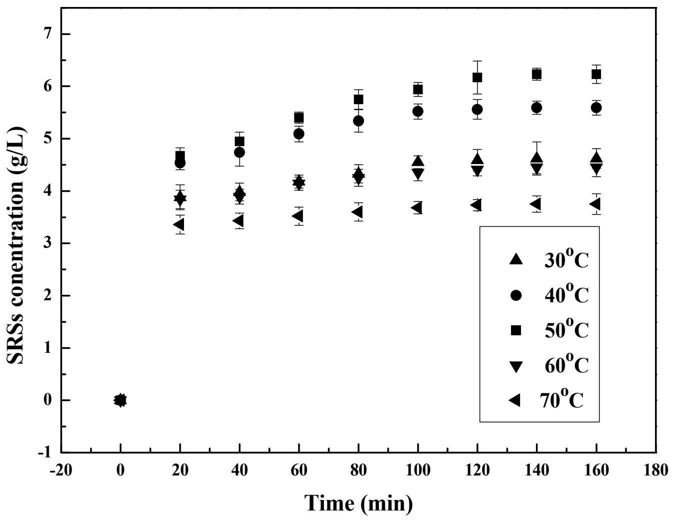

Figure C. Enzymes kinetics.

C.1. Pepsin

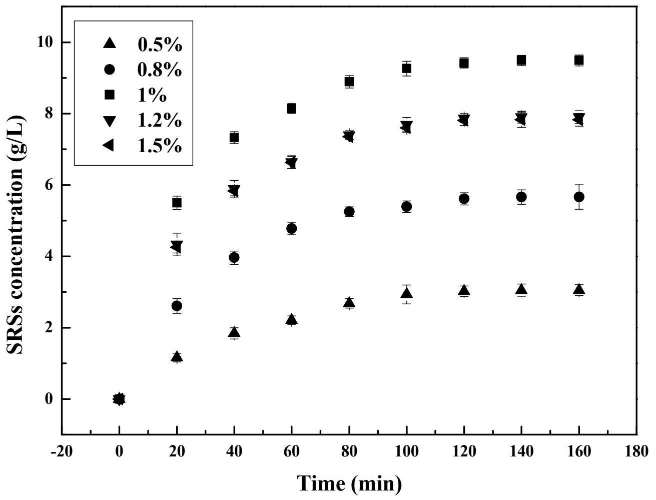

C.2. Chitosanase

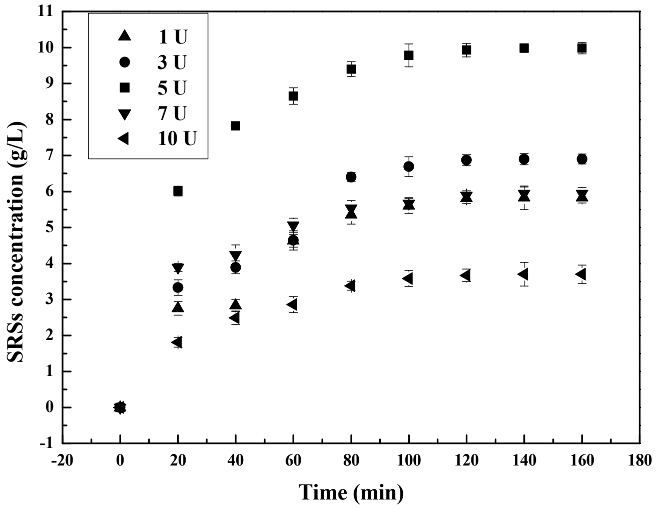

C.3. α-amylase

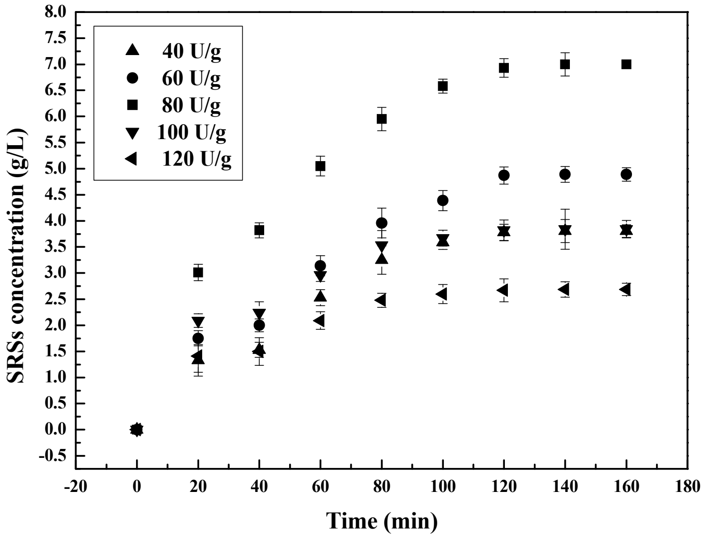

Text S1: Relationship between pH and degradation mechanisms at the active site of each enzyme.

1. The two major mechanisms of enzymatic glycosidic bond hydrolysis by chitosanases: Retaining mechanism (a) in which the glycosidic oxygen is protonated by Glu22-H and nucleophilic assistance to aglycon departure is provided by Asp40 (vice versa) and inverting mechanism (b) in which protonation of the glycosidic oxygen and aglycon departure are accompanied by a concomitant attack of a water molecule that is activated by general base residue B [38].
2. Mechanism proposed by Andreeva and Rumsh Part A represents the free enzyme, with hydrogen bonds from the Asp groups to the bound water molecule (W1) and no hydrogen bond between the two Asps: A hydrogen bond from Thr 218 to Asp 215 keeps the Asp in the negatively-charged form, and this residue acts as the general base to remove a proton from the water molecule (W1) during attack on a carbonyl group. Furthermore, the second water molecule (W2) is hydrogen bonded to the -OH of Ser 35 and the carbonyl oxygen of Asn 37. Following the approach of the peptide to be cleaved, the hydrogen bond from Thr 218 to Asp 215 is broken so that Asp 215 can take a proton from W1, which attacks the carbonyl (Part B). The outer oxygen of Asp 32 donates a proton to the carbonyl group of the peptide bond, while the -OH of Ser 35 moves to form a new hydrogen bond to the oxygen of Asp 32. Water molecule W2 changes the hydrogen-bonding pattern to donate a hydrogen bond to the oxygen of the side chain of Ser 35 while accepting a hydrogen bond from Tyr 75. The formation of a new hydrogen bond from Trp 39 to the side chain oxygen of Tyr 75 facilitates the donation of the new hydrogen bond to W2. The new hydrogen bonds of Tyr 75 and Trp 39 are formed following the movement of the flap to bring Tyr 75 closer to both the W2 and the Trp. In the final steps of this mechanism, Asp 215 will give up a proton to the nitrogen of the leaving group, and Asp 32 will take a proton away from the diol intermediate to create the electron push necessary to break the carbonyl carbon to nitrogen bond. Reformation of the hydrogen bonds with Thr 218 to Asp 215 and Ser 35 with W2 returns the structure to that seen in Part A. [58].
3. Catalytic steps in glycoside bond cleavage in retaining enzymes: The proton donor protonates the glycosidic oxygen, and the catalytic nucleophile attacks at C1, leading to the formation of the first transition state. The catalytic base promotes the attack of the incoming molecule ROH (water in hydrolysis or another sugar molecule in transglycosylation) on the formation of the covalent intermediate, resulting in a second transition state, leading to hydrolysis or the transglycosylation product [54].
